# Determination of insulin therapy perceptions in patients with diabetes mellitus and prediabetes attending the diabetes outpatient clinic

**DOI:** 10.1186/s12902-026-02205-1

**Published:** 2026-02-24

**Authors:** Ahmet Cakir, Hasan Memis, Nesligül Ozdemir-Ayduran, Serenay Tarin, Bahri Evren

**Affiliations:** 1 Department of Clinical Pharmacy, Faculty of Pharmacy, Inonu University, Malatya, Türkiye; 2 Deparment of Clinical Pharmacy, Faculty of Pharmacy, Afyonkarahisar Health Sciences University, Afyonkarahisar, Türkiye; 3 Department of Clinical Pharmacy, Faculty of Pharmacy, Gazi University, Ankara, Türkiye; 4 Faculty of Pharmacy, Inonu University, Malatya, Türkiye; 5 Department of Endocrinology, Faculty of Medicine, Inonu University, Malatya, Türkiye

**Keywords:** Diabetes mellitus, Insulin, Insulin therapy perception, Prediabetic state

## Abstract

**Background:**

Negative perceptions of insulin therapy are common in diabetes mellitus (DM) patients and may delay treatment. Insulin perception has been studied in type 2 diabetics but not type 1 or prediabetics. The aim of this study was to determine the differences in perceptions of insulin use between diabetes types.

**Methods:**

This cross-sectional study was conducted at a tertiary care hospital between December 2023 and April 2024. Participants were adult diabetes outpatient clinic patients with T1DM, T2DM, or prediabetes. The validated Insulin Therapy Appraisal Scale (ITAS) quantified patients’ insulin therapy perceptions. Patients who did not use insulin and were in the prediabetes category answered the questions based on their current knowledge about insulin treatment. Higher scores on the positive sub-dimension indicate more favorable perceptions of insulin therapy, whereas higher scores on the negative sub-dimension and total ITAS reflect more negative perceptions. *p*-value < 0.05 was considered statistically significant.

**Results:**

A total of 160 adult patients were included in the study. Patients with T1DM demonstrated significantly higher ITAS positive sub-dimension scores and lower negative sub-dimension and total ITAS scores compared with patients with T2DM and prediabetes (*p* < 0.05). No significant differences in ITAS scores were observed between the T2DM and prediabetes groups. Correlation analyses showed that the ITAS positive sub-dimension score was inversely associated with age and C-peptide levels. In contrast, negative sub-dimension and total ITAS scores were positively correlated with age and C-peptide levels but negatively correlated with diabetes duration. Marital status, highest education level, and regular blood glucose monitoring were found to significantly influence insulin perception (*p* < 0.05). Female patients exhibited more positive perceptions, whereas insulin users, patients without diabetes-related complications, and those who exercised regularly demonstrated less negative attitudes toward insulin therapy.

**Conclusion:**

Patients with T1DM have significantly more positive perceptions of insulin therapy compared with patients with T2DM and prediabetes. Identifying factors influencing insulin-related perceptions may help improve acceptance of insulin therapy and enhance treatment adherence in patients with diabetes.

**Clinical trial number:**

N/A.

## Background

Diabetes Mellitus (DM) is a multifaceted, chronic disorder necessitating continuous medical attention and comprehensive risk mitigation techniques in addition to glucose control. Continuous diabetes self-management education and support is essential to empower individuals, avert acute problems, and diminish the risk of long-term complications. There is considerable evidence to endorse several therapies aimed at enhancing DM outcomes [1]. Patients with Type 2 Diabetes Mellitus (T2DM) are typically commenced on oral antidiabetic medications, physical activity, and dietary modifications; but, as the condition advances, the majority of patients ultimately necessitate insulin therapy to sustain glycemic regulation [2]. For the patient to derive benefits from insulin therapy, it is crucial that they accept and adhere to the treatment, which is facilitated through education and information dissemination [3]. Medications are efficacious solely when administered in accordance with healthcare experts’ recommendations; nevertheless, inadequate adherence among diabetes patients persists as a prevalent issue [4].

In clinical practice, the commencement of treatment is frequently postponed due to several factors, including patients’ unwillingness to embrace insulin therapy. These factors include the difficult and complex nature of insulin therapy, the concerning painful injections, the risk of hypoglycemia, and the anticipated weight gain [5]. Negative perceptions regarding insulin utilization may lead patients to resist the beginning of insulin therapy [6]. Holmes-Truscott et al. reported that adults with T2DM who were not using insulin had significantly higher total ITAS scores compared with insulin users, reflecting more negative appraisals of insulin therapy [7]. Similarly, Farsaei et al. reported that insulin omission and low adherence were important contributors to inadequate glycemic control among patients with diabetes [4]. Furthermore, the systematic review and meta-analysis by Gimeno et al. reported that poor adherence and low persistence to basal insulin therapy in individuals with T2DM were consistently associated with poorer glycemic outcomes across European studies [8]. These findings indicate that insulin-related perceptions may directly influence metabolic outcomes, underscoring the clinical importance of evaluating such attitudes across different stages of glucose dysregulation. The authors further noted that negative insulin perceptions were closely linked to reluctance toward insulin initiation and psychological insulin resistance. It has been observed that studies evaluating attitudes towards insulin treatment in literature were conducted only in individuals with T2DM [7, 9, 10]. However, it is also important to determine the attitudes of patients with T1DM and prediabetes towards insulin therapy. Since insulin use is the only treatment option for patients with Type 1 Diabetes Mellitus (T1DM), it is known that the perception of insulin treatment affects these patients more than patients with T2DM. Furthermore, the difficulty patients face in titrating their insulin dose is the primary factor contributing to this situation [11]. Studies have shown that early identification of the attitude towards disease risk in people with prediabetes reduces the risk of disease progression [12].

To the best of our knowledge, this is the first study to evaluate insulin therapy perceptions using the Insulin Therapy Appraisal Scale simultaneously in patients with T1DM, T2DM, and prediabetes. In addition, by examining the association between insulin perceptions and C-peptide levels, this study provides novel insights into the physiological determinants of insulin-related attitudes. Furthermore, the inclusion of individuals with prediabetes offers an opportunity to identify negative perceptions at an early stage, which may help predict future acceptance of insulin therapy and guide timely educational and clinical interventions.

This study compared insulin therapy perceptions in T1DM, T2DM, and prediabetes patients and examined the link between insulin-related attitudes and sociodemographic and clinical variables. The study examined the relationship between physiological indicators like C-peptide levels and disease duration and insulin perceptions to better understand how insulin attitudes change during glucose dysregulation and how they may affect future treatment acceptance.

## Methods

### Ethics committee approval

Ethical approval for the study was obtained from Inonu University on 28 November 2023, with approval number 2023/5236.

### Study participants

Patients who referred to the diabetes ambulatory clinic of a university affiliated tertiary care hospital between December 2023 and April 2024 and fulfilled the following criteria were included in this cross-sectional study: being at least 18 years of age by the time they referred, giving consent to participate in the study, and being diagnosed with prediabetes, T1DM or T2DM.

T1DM is defined as insulin-dependent diabetes mellitus, while T2DM is defined as non-insulin-dependent diabetes mellitus. Prediabetes was defined by a fasting glucose level of 100 to 125 mg/dL, a glucose level of 140 to 199 mg/dL measured 2 h after a 75 g oral glucose load, or a glycated hemoglobin rate (HbA1C) of 5.7% to 6.4% [13]. This information has been validated by clinicians.

The Insulin Therapy Appraisal Scale (ITAS), evaluated for validity and reliability by Sürücü et al. in a Turkish study, was utilized to assess patients’ perceptions of insulin use [5]. Before or after outpatient clinic visits, patients were interviewed individually outside the clinic. Individual interviews prevented patients from being impacted by each other. A clinical pharmacist administered the scale to the patients. Patients without insulin and those with prediabetes answered questions depending on their existing insulin knowledge. Illiterate patients were interviewed in person with the researcher reading questions aloud. Survey questions were provided to patients without annotations or remarks to eliminate bias.

### Insulin therapy appraisal scale

The Insulin Therapy Appraisal Scale (ITAS), a 20-item instrument originally developed by Snoek et al., was utilized to evaluate the patients’ perceptions of insulin treatment [14]. The scale is used to examine the experiences of insulin users as well as the expectations of non-users regarding insulin use. The scale was adapted into Turkish language and culture and validated by Surucu et al. [5]. Written consent to use the Turkish scale was obtained from the corresponding author *via* email. This scale employed a five-point Likert scale, ranging from “Absolutely disagree (1 point)” to “Absolutely agree (5 points)”. The final Turkish scale consisted of two sub-dimensions, namely positive sub-dimension (item number 3, 8, 17, and 19) and negative sub-dimension (item number 1, 2, 4, 5, 6, 7, 9, 10, 11, 12, 13, 14, 15, 16, 18, and 20). The total sum of positive sub-dimension item scores indicates a positive perception, while the sum of negative sub-dimension item scores and the total scale score indicate a negative perception regarding insulin treatment. The total scale score is calculated by reversing the positive sub-dimension item scores and adding this to the sum of the negative sub-dimension item scores. The scale has no cut off value. The final Turkish scale has an internal consistency of Cronbach alpha coefficient of 0.80, 0.64, and 0.83 for whole scale, positive, and negative sub-dimensions, respectively [5].

The questionnaire comprised four parts. The first part obtained written consent from patients to participate. The second part collected sociodemographic data (age, sex, highest education level, family structure, employment status, marital status, and residence) and anthropometric measurements (body weight, height, and body mass index). The third part gathered disease-related information, including diabetes duration, current HbA1C, current C-peptide levels, the count of diabetes-related complications, comorbidities, hospitalizations in the last year, regular blood glucose monitoring, and insulin usage. The final part consisted of the ITAS. The questionnaire was administered to the participant face-to-face using Google Forms by two senior pharmacy students.

### Sample size calculation

Sample size calculation was performed using G*Power version 3.1.9.6 (Heinrich Heine University, Düsseldorf, Germany). Because median ITAS scores were compared among prediabetes, T1DM, and T2DM patient groups, the ANOVA: Fixed effects omnibus one-way test was selected. The required sample size was calculated as 159, assuming an alpha error of 0.05, an effect size of 0.25 [corresponding to a medium effect size [15] ], and a power of 0.80.

### Statistical analysis

The statistical analysis was performed using Statistical Package for the Social Sciences for Windows^®^ version 27.0.1 (IBM Corp, Chicago, IL, USA). Categorical variables were given in number (percentage), and continuous variables were given in median [interquartile range (IQR)]. Normality of the continuous variables was tested using Kolmogorov-Smirnov test and none of them showed normal distribution, hence non-parametric tests were used in hypothesis testing. Distribution of categorical variables was tested using χ^2^ test. The medians of the continuous variables were compared using Mann-Whitney U test or Independent-Samples Kruskal-Wallis test if they consisted of two levels or more, respectively. For pairwise comparisons of continuous variables showing statistical significance in the Kruskal-Wallis test, the Bonferroni test was applied as a *post-hoc* analysis. In correlation analysis, Spearman test was used. A *p*-value < 0.05 was considered as statistically significant.

## Results

The study included 160 adult patients, 68 of which are T1DM, 62 of which is T2DM, and 30 of which is prediabetic. The patients’ sociodemographic, anthropometric, and disease-related data is summarized in Table 1.

**Table 1:** Characteristics of the patients

Characteristics	T1DM^‡^	T2DM^§^	Prediabetes	Total	*p*
**Sex**,** n (%)**					0.162^a^
Male	26 (38.24)	32 (47.06)	10 (14.71)	68 (42.50)	
Female	42 (45.65)	30 (32.61)	20 (21.74)	92 (57.50)	
**Marital status**,** n (%)**					NR
Single	42 (85.71)	4 (8.16)	3 (6.12)	49 (30.63)	
Married	25 (23.58)	54 (50.94)	27 (25.47)	106 (66.25)	
Widow/Divorced	1 (20.00)	4 (80.00)	0 (0)	5 (3.13)	
**Highest education level**,** n (%)**					NR
Primary school	3 (6.38)	34 (72.34)	10 (21.28)	47 (29.38)	
Middle school	3 (21.43)	7 (50.00)	4 (28.57)	14 (8.75)	
High school	27 (50.00)	15 (27.78)	12 (22.22)	54 (33.75)	
Bachelor’s degree	32 (78.05)	6 (14.63)	3 (7.32)	41 (25.62)	
Postgraduate	3 (75.00)	0 (0)	1 (25.00)	4 (2.50)	
**Employment status**,** n (%)**					0.217^a^
Employed	24 (45.28)	16 (30.19)	13 (24.53)	53 (33.13)	
Unemployed	44 (41.12)	46 (42.99)	17 (15.89)	107 (66.88)	
**Regular exercise status**,** n (%)**					**0.003** ^**a**^
Yes	33 (61.11)	15 (27.78)	6 (11.11)	54 (33.75)	
No	35 (33.02)	47 (44.34)	24 (22.64)	106 (66.25)	
**Regular blood glucose monitor status**,** n (%)**					**< 0.001** ^**a**^
Yes	46 (64.79)	21 (29.58)	4 (5.63)	71 (44.38)	
No	22 (24.72)	41 (46.07)	26 (29.21)	89 (55.63)	
**Residence**,** n (%)**					**0.030** ^**a**^
Village	4 (22.22)	13 (72.22)	1 (5.56)	18 (11.25)	
District	17 (51.52)	9 (27.27)	7 (21.21)	33 (20.63)	
City	47 (43.12)	40 (36.70)	22 (20.18)	109 (68.13)	
**Being insulin user**,** n (%)**					**< 0.001** ^**a**^
Yes	68 (60.18)	45 (39.82)	0 (0)	113 (70.63)	
No	0 (0)	17 (36.17)	30 (63.83)	47 (29.38)	
**Age**,** years**,** median [IQR]**	27.00 [21.00–35.00]	59.50 [50.50–65.00]	46.50 [39.00–51.50]	43.50 [28.00–58.00]	**< 0.001** ^**b**^
**Height**,** cm**,** median [IQR]**	167.00 [160.00–175.75]	165.00 [155.75–171.25]	165.00 [160.00–172.50]	167.00 [159.00–172.75]	0.130^b^
**Weight**,** kg**,** median [IQR]**	70.00 [58.00–78.00]	80.00 [68.00–90.00]	79.00 [69.50–93.00]	75.00 [65.00–87.75]	**< 0.001** ^**b**^
**Body mass index**,** kg/m**^**2**^, **median [IQR]**	23.98 [21.88–27.78]	28.73 [25.89–32.90]	28.60 [24.57–34.16]	27.21 [23.24–30.79]	**< 0.001** ^**b**^
**Diabetes duration**,** years**,** median [IQR]**	12.00 [8.00–17.00]	7.00 [4.00–14.00]	1.00 [0–1]	8.00 [2.00–15.00]	**< 0.001** ^**b**^
**HbA1C**^†^, **%**,** median [IQR]**	8.50 [7.45–10.00]	9.30 [6.90–12.20]	5.90 [5.78–6.20]	8.10 [6.33–10.10]	**< 0.001** ^**b**^
**C-peptide**,** ng/mL**,** median [IQR]**	0.15 [0.11–0.25]	1.96 [1.52–2.68]	2.54 [1.93–3.57]	1.26 [0.15–2.34]	**< 0.001** ^**b**^
**Count of diabetes-related complications**,** median [IQR]**	0 [0–0]	1.00 [0–1.00]	0 [0–0]	0 [0–1.00]	**< 0.001** ^**b**^
**Count of comorbidities**,** median [IQR]**	0 [0–1.00]	0 [1.00–2.00]	0 [1.00–2.00]	1 [0–1.00]	**< 0.001** ^**b**^
**Count of hospitalizations in the last year**,** median [IQR]**	0 [0–1.00]	1.00 [0–1.00]	0 [0–0]	0 [0–1.00]	**< 0.001** ^**b**^

^†^HbA1C: Glycated hemoglobin rate

^‡^T1DM: Type 1 diabetes mellitus

^§^T2DM: Type 2 diabetes mellitus

IQR: Interquartile range

NR: Not reported since at least 20% of the cells have expected count less than 5

^a^ χ^2^ test

^b^ Independent-Samples Kruskal-Wallis Test

Age was significantly different between T1DM and T2DM (*p* < 0.001), T1DM and Prediabetes (*p* < 0.001), and Prediabetes and T2DM (*p* = 0.003).

Weight varied significantly between T1DM and T2DM (*p* = 0.001) and T1DM and Prediabetes (*p* = 0.003). Body mass index (BMI) showed significant differences between T1DM and T2DM (*p* < 0.001) and T1DM and Prediabetes (*p* < 0.001).

Diabetes duration was significantly different between T1DM and T2DM (*p* = 0.035), T1DM and Prediabetes (*p* < 0.001), and Prediabetes and T2DM (*p* < 0.001).

HbA1C levels were significantly different between T1DM and Prediabetes (*p* < 0.001) and between Prediabetes and T2DM (*p* < 0.001).

C-peptide levels varied significantly between T1DM and T2DM (*p* < 0.001) and T1DM and Prediabetes (*p* < 0.001).

The count of diabetes-related complications differed significantly between T1DM and T2DM (*p* < 0.001) and Prediabetes and T2DM (*p* < 0.001).

The number of comorbidities was significantly different between T1DM and T2DM (*p* < 0.001) and T1DM and Prediabetes (*p* = 0.001). The count of hospitalizations in the last year was significantly different between T1DM and Prediabetes (*p* = 0.001) and Prediabetes and T2DM (*p* < 0.001).

The comorbidities of the patients are summarized in Table 2.

**Table 2:** Comorbidities of the patients

Comorbidity	ICD-10 Code^£^	T1DM^‡^, *n* (%)	T2DM^§^, *n* (%)	Prediabetes, *n* (%)	Total, *n* (%)
Essential hypertension	I10	9 (13.24)	25 (40.32)	11 (36.67)	45 (28.13)
Chronic ischemic heart disease	I25	1 (1.47)	12 (19.35)	1 (3.33)	14 (8.75)
Hyperlipidemia, unspecified	E78.5	0 (0)	8 (12.90)	4 (13.33)	12 (7.50)
Other hypothyroidism	E03	3 (4.41)	6 (9.68)	2 (6.67)	11 (6.88)
Asthma	J45	1 (1.47)	3 (4.84)	1 (3.33)	5 (3.13)
Other		19 (27.93)	20 (32.24)	13 (43.31)	52 (32.60)
None	-	47 (69.12)	16 (25.81)	8 (26.67)	71 (44.38)

^£^ICD-10: International Classification of Diseases, Tenth Revision

^‡^T1DM: Type 1 diabetes mellitus

^§^T2DM: Type 2 diabetes mellitus

The median ITAS total scores and subdimension scores were compared based on patient-related factors. The results are given in Table 3.

**Table 3:** Comparison of ITAS scores among patient groups based on patient-related factors

Characteristics	ITAS^¶^ positive subdimension score, median [IQR]	*p*	ITAS^¶^ negative subdimension score, median [IQR]	*p*	ITAS^¶^ total score, median [IQR]	*p*
**Sex**						
Male	13.00 [12.00–15.00]	**0.001** ^a^	45.00 [39.00–52.00]	0.176^a^	55.50 [48.00–64.00]	0.072^a^
Female	14.00 [12.50–16.00]		44.00 [36.00–50.00]		52.00 [44.00–61.00]	
**Marital status**						
Single	15.00 [14.00–16.00]	**0.018** ^b^	38.00 [31.00–44.00]	**< 0.001** ^b^	48.00 [41.00–54.00]	**< 0.001** ^b^
Married	13.00 [12.00–15.00]		47.00 [40.00–52.00]		57.00 [50.00–64.00]	
Widow/Divorced	14.00 [12.00–17.00]		44.00 [42.00–48.00]		54.00 [50.00–58.00]	
**Highest education level**						
Primary school	12.00 [12.00–15.00]	**0.004** ^b^	49.00 [44.00–54.00]	**< 0.001** ^b^	61.00 [54.50–66.00]	**< 0.001** ^b^
Middle school	14.00 [12.00–15.00]		45.00 [39.00–52.00]		55.00 [49.00–61.00]	
High school	14.00 [12.00–16.00]		42.50 [35.00–50.00]		54.00 [44.00–60.00]	
Bachelor’s degree	14.00 [13.00–16.00]		42.00 [36.00–47.00]		50.00 [45.00–57.00]	
Post graduate	17.50 [15.50–18.50]		27.50 [21.00–35.50]		35.00 [26.50–44.00]	
**Employment status**						
Employed	14.00 [12.00–15.00]	0.940^a^	44.00 [38.00–50.00]	0.487^a^	52.00 [46.00–60.00]	0.442^a^
Unemployed	14.00 [12.00–15.50]		45.00 [37.00–51.00]		55.00 [47.00–62.00]	
**Regular exercise status**						
Yes	14.00 [12.00–16.00]	0.171^a^	40.50 [35.00–47.00]	**0.015** ^**a**^	51.50 [43.00–57.00]	**0.014** ^a^
No	14.00 [12.00–15.00]		46.00 [38.00–52.00]		56.00 [48.00–64.00]	
**Regular blood glucose monitor status**						
Yes	14.00 [12.50–16.00]	**0.035** ^**a**^	43.00 [35.00–48.00]	**0.044** ^**a**^	52.00 [43.50–58.50]	**0.027** ^**a**^
No	14.00 [12.00–15.00]		46.00 [39.00–52.00]		56.00 [49.00–64.00]	
**Residence**						
Village	14.00 [11.00–15.00]	0.652^b^	46.00 [38.00–52.00]	0.059^b^	54.50 [49.00–65.00]	0.072^b^
District	14.00 [12.00–15.00]		47.00 [40.00–52.00]		57.00 [51.00–64.00]	
City	14.00 [12.00–16.00]		44.00 [36.00–50.00]		53.00 [45.00–61.00]	
**Being insulin user**						
Yes	14.00 [12.00–16.00]	0.051^a^	44.00 [35.00–48.00]	**0.004** ^**a**^	53.00 [45.00–60.00]	**0.003** ^**a**^
No	13.00 [12.00–15.00]		50.00 [39.50–54.50]		61.00 [50.50–65.50]	
**Diabetes related complications** ^**#**^						
Yes	14.00 [12.00–16.00]	0.321	47.00 [40.00–51.00]	**0.018**	56.00 [50.00–64.00]	**0.017**
No	14.00 [12.00–15.00]		41.00 [36.00–50.00]		52.00 [44.00–60.00]	
**Diabetes type**						
T1DM^‡^	15.00 [13.25–16.00]	**0.002** ^**b**^	38.00 [32.00–46.00]	**< 0.001** ^**b**^	48.00 [41.25–55.00]	**< 0.001** ^**b**^
T2DM^§^	13.00 [12.00–15.00]		48.50 [44.00–54.00]		60.00 [54.00–64.50]	
Prediabetes	12.00 [11.75–15.00]		46.00 [38.00–54.25]		58.00 [48.50–66.00]	
All patients	14.00 [12.00–15.75]		44.00 [37.00–51.00]		54.00 [47.00–62.00]	

^¶^ITAS: Insulin treatment appraisal scale

^‡^T1DM: Type 1 diabetes mellitus

^§^T2DM: Type 2 diabetes mellitus

IQR: Interquartile range

^a^ Independent-Samples Mann-Whitney U Test

^b^ Independent-Samples Kruskal-Wallis Test

^**#**^Retinopathy, neuropathy, nephropathy, diabetic foot, or diabetic ketoacidosis

Pairwise comparisons of ITAS scores using Bonferroni *post-hoc* test among different patient groups based on marital status and education levels revealed significant differences in several cases. Married individuals had significantly different ITAS scores compared to single individuals in the positive subdimension (*p* = 0.015), negative subdimension (*p* < 0.001), and total score (*p* < 0.001). Regarding education levels, primary school graduates showed significant differences in ITAS scores compared to bachelor’s degree holders (*p* = 0.019 for positive, *p* = 0.012 for negative, *p* = 0.002 for total score) and postgraduates (*p* = 0.034 for positive, *p* = 0.008 for negative, *p* = 0.003 for total score). Additionally, primary school graduates had significantly different scores compared to high school graduates in the negative subdimension (*p* = 0.009) and total score (*p* = 0.006). All *p*-values were Bonferroni corrected for statistical significance.

ITAS positive subdimension scores varied significantly between T1DM and T2DM (*p* = 0.008) and T1DM and Prediabetes (*p* = 0.017). ITAS negative subdimension scores were significantly different between T1DM and T2DM (*p* < 0.001) and T1DM and Prediabetes (*p* = 0.003). ITAS total scores also showed significant differences between T1DM and T2DM (*p* < 0.001) and T1DM and Prediabetes (*p* = 0.002).

We performed Spearman’s correlation analysis to reveal the relationship between ITAS subdimensions and diabetes-related parameters. The results are shown in Table 4.

**Table 4:** Correlation between ITAS scores and diabetes-related parameters

Factor pairs	*r*	*p* ^a^
ITAS^¶^ positive subdimension score - Age	-0.226	**0.004**
ITAS^¶^ negative subdimension score -Age	0.404	**< 0.001**
ITAS^¶^ total score-Age	0.411	**< 0.001**
ITAS^¶^ positive subdimension score - Diabetes duration		0.181
ITAS^¶^ positive subdimension score - HbA1C^†^		0.431
ITAS^¶^ positive subdimension score - C-peptide	-0.259	**< 0.001**
ITAS^¶^ negative subdimension score - Diabetes duration	-0.230	**0.003**
ITAS^¶^ negative subdimension score - HbA1C^†^		0.323
ITAS^¶^ negative subdimension score - C-peptide	0.439	**< 0.001**
ITAS^¶^ total score - Diabetes duration	-0.225	**0.004**
ITAS^¶^ total score - HbA1C^†^		0.272
ITAS^¶^ total score - C-peptide	0.439	**< 0.001**

^†^HbA1C: Glycated hemoglobin rate

^¶^ITAS: Insulin treatment appraisal scale

^a^Spearman’s correlation test

## Discussion

This study aimed to compare the perceptions of prediabetes, T1DM, and T2DM patients regarding the use of insulin therapy and to determine the factors affecting this perception. Identifying diabetes patients’ insulin treatment approaches will help determine their acceptance of the treatment at diagnosis or along the disease process.

This study covered 160 patients. The groups differed in age, weight, body mass index, exercise status, insulin use, and blood sugar monitoring, but not in gender or employment position. As expected, T1DM were younger, thinner, had lower body mass index, and were more likely to monitor blood glucose levels. Additionally, compared to other groups, T1DM individuals had lower C-peptide levels and longer durations of diabetes.

Diabetes-related complications were less common in patients with T1DM than in those with T2DM. T1DM patients had hemoglobin A1c levels that were lower than those of T2DM patients, even though there was no statistically significant difference between the two groups. T1DM patients may have better blood glucose control, resulting in fewer diabetes complications than T2DM patients. T1DM patients were younger and had less chronic conditions.

Prediabetes patients were generally similar to T2DM patients in terms of weight, body mass index, C-peptide levels and comorbidities. Patients with prediabetes were younger, had lower hemoglobin A1c levels, and experienced fewer diabetes-related complications and associated hospitalizations than those with T2DM. It is well recognized that the risk of T2DM in the future is extremely high if prediabetes is not identified and treated promptly. In a study conducted in women and men diagnosed with prediabetes at the age of 45 according to the American Diabetes Association’s criteria, the risk of developing T2DM within 10 years was found to be 14.2% and 9.2%, respectively [16]. The individuals in our study who had prediabetes shared traits with those who had T2DM, which is a strong sign that prediabetes will develop into diabetes if precautions and treatment are delayed.

When we analyzed our study’s primary finding, we discovered that T1DM patients’ perceptions of insulin therapy were significantly more positive than those of prediabetes and T2DM patients. Compared to individuals with T2DM and prediabetes, those with T1DM showed statistically greater ITAS positive subdimension scores and lower total and ITAS negative subdimension scores. All patients with T1DM were using insulin. Therefore, they were more familiar with insulin treatment. This may have led to fewer fears about treatment and less negative attitudes towards treatment. When the literature is examined, studies evaluating ITAS scores mostly in individuals with T2DM stand out.

In a study by Özkan et al. evaluating the perception of insulin treatment in patients with T2DM, the mean scores of the patients were found to be 14.1 ± 4.1, 47.3 ± 12.9 and 57.1 ± 11.6 for the positive subdimension ITAS, negative subdimension ITAS and total ITAS, respectively [17]. In another study conducted in Türkiye, the mean ITAS total score of T1DM and T2DM patients receiving insulin treatment in hospital was found to be 56.25 ± 0.819 [18].

In a study conducted by Woudenberg et al., the mean Total ITAS score of patients with T2DM was found to be 37 ± 9 [19].

Holmes-Truscott et al. evaluated the ITAS score in T2DM patients who used and did not use insulin. The results showed that the difference between the groups was statistically significant, with the score of those who used insulin being 50.2 ± 10.3 and the score of those who did not use insulin being 60.7 ± 10.1. Those who utilized insulin were shown to have a more positive perception [7].

When comparing our study with the studies in literature, it is seen that the ITAS scores of individuals with T2DM are similar. Both in our study and in studies in literature, it was observed that patients with T2DM had a highly negative attitude towards insulin treatment. Compared with Woudenberg’s study, our patients appear to have higher ITAS scores. This difference may be due to the diversity of patients’ demographic characteristics.

The ITAS score is typically assessed in individuals with T2DM rather than those with T1DM and prediabetes, according to the literature review. To the best of our knowledge, our study is the first to assess the ITAS score in patients with T1DM and prediabetes as well as those with T2DM.

The attitudes and behaviors of patients with T1DM and T2DM towards insulin treatment may differ from each other. Although there is no study directly examining the attitudes of T1DM and T2DM patients towards insulin treatment, when treatment adherence is considered, it is seen that T2DM patients are more non-adherent. In a study by Chefik and his colleagues evaluating adherence to insulin treatment, it was determined that individuals with T2DM were 77% times less likely to adhere to insulin treatment compared to individuals with T1DM [20]. A systematic review revealed that nearly half of patients with T2DM in Europe did not adhere to insulin therapy, and one-third discontinued treatment [8].

Low treatment adherence is a major factor in insulin treatment’s negative perception. The reasons for patients’ low adherence to insulin treatment are mostly fear of needles, treatment-related weight gain, side effects such as hypoglycemia, and difficulties in injecting [21]. Apart from these factors, the duration of diabetes is also a significant factor influencing adherence to insulin treatment. One study examining the reasons for low adherence rates to insulin treatment in patients with T2DM found that obesity, polypharmacy, advanced age, and short diabetes duration were the significant causes of non-adherence [22]. The short duration of diabetes may contribute to non-adherence, as patients may not yet fully grasp the severity of the disease or not feel concerned about their condition due to the absence of complications. In our study, individuals with T1DM who had been living with the disease for a long time showed a more favorable attitude about insulin treatment than patients with T2DM.

In our study, when the other parameters that differed in terms of ITAS positive subdimension scores were examined, younger age, female gender, being single, having a higher level of education, and monitoring blood sugar levels were associated with higher ITAS positive subdimension scores. On the other hand, those who were older, male patients, those who were married or divorced, had low educational status level, did not exercise regularly, did not measure their blood glucose regularly, or did not use insulin, had diabetes related complications were found to have high ITAS negative subdimension and total ITAS scores, which are a sign of a negative perception to insulin treatment. Similar to our study, Ku et al.‘s study shows that women have higher positive attitudes on insulin treatment [23]. Higher education may improve perception because of better awareness of the condition and its treatment. Awareness may lead to a better informed and positive approach to managing the disease.

Significant disparities in attitudes between insulin users and non-users were found when the characteristics linked to differences in ITAS scores were evaluated in the literature. In the study by Holmes-Truscott et al., the total ITAS score was found to be higher in those who did not use insulin [7]. Similarly, in the study conducted by Ismailoglu et al., it was observed that insulin users had higher ITAS positive subdimension scores and lower ITAS negative subdimension scores [24]. In our study, similar to these studies, it was determined that individuals using insulin had a more positive attitude towards insulin.

In our study, the ITAS score was assessed not only based on insulin use but also, unlike previous studies, in relation to its correlation with C-peptide levels. While ITAS positive subdimension scores showed a weak negative correlation with C-peptide levels, ITAS negative subdimension scores and ITAS total scores showed a moderate positive correlation with c-peptide levels. As C-peptide levels increase, the ITAS positive subgroup scores decrease and ITAS negative subdimension scores and ITAS total score increase. Clinical evaluation of this result reveals that individuals with significant C-peptide reserve (i.e., T2DM and prediabetes) have lower ITAS positive scores and higher ITAS negative scores. Our results, which show that T1DM have high ITAS positive scores and low total and negative subdimension ITAS scores, support this conclusion.

A weak negative correlation between diabetes duration and both the ITAS negative score and the ITAS total score was discovered when the association between diabetes duration and ITAS scores was investigated. In our study, patients with T1DM had longer-term diabetes compared to other patients. In contrast to patients with T2DM and prediabetes, this data demonstrates that individuals with T1DM have a more positive attitude about insulin treatment. Unlike our study, Holmes et al. did not find any correlation with the duration of diabetes [7]. In our study, individuals with T1DM exhibited a longer duration of diabetes compared to those with T2DM, resulting in a statistically significant difference in diabetes duration. In contrast, the Holmes study, which exclusively included patients with T2DM, reported a more homogenous distribution of diabetes durations. As a consequence, this similarity in diabetes duration did not contribute to any significant variation in ITAS scores.

In Holmes’s study, age showed a weak positive correlation [7]. Similar to Holmes’ study, in our study, total and negative ITAS scores showed a weak positive correlation with age, while positive ITAS scores showed a negative correlation. However, while Holmes looked at the correlation in T2DM patients who used and did not use insulin, we included T1DM, 2 diabetes and prediabetes patients in our study. Thus, the effect of age on diabetic patients was evaluated in a larger population. As a result, it can be said that with increasing age, a negative perception of insulin use occurs in patients.

One of the prominent findings of our study is that individuals with prediabetes had similar negative attitudes towards insulin treatment as individuals with T2DM. We believe that determining ITAS scores in individuals with prediabetes in advance is important to prevent patients from delaying their treatment in the following years. It will be possible to prevent negative perceptions about insulin treatment and increase compliance with treatment through education and counseling services for individuals with negative ITAS scores.

It could be suggested that the more favorable insulin perceptions observed among patients with T1DM are not surprising, considering their long-standing experience with insulin therapy. However, our aim was not to evaluate the immediate psychological impact of starting insulin, but rather to explore how insulin-related perceptions differ across various stages of dysglycemia within a real-world clinical setting. Including patients with established T1DM reflects routine outpatient practice and strengthens the external validity of our findings. Limiting the sample to newly diagnosed T1DM patients or excluding individuals with T2DM who use insulin would have reduced the representativeness of the study population and restricted a meaningful comparison across the full disease spectrum. Thus, while the overall direction of the findings may seem clinically intuitive, systematically quantifying these differences and demonstrating their relationship with physiological markers such as C-peptide levels offers novel and clinically relevant insight into how insulin perceptions change throughout the progression of glucose dysregulation.

This study has some limitations. Patient characteristics and insulin therapy perceptions were collected using self-reports. Social desirability bias and recollection bias could reduce data accuracy. Selection bias may have occurred because participants were from a diabetes clinic. Compared to individuals who did not seek care, patients who visited the clinic can have different perspectives and experiences about diabetes and insulin treatment. The study focused on demographic and medical factors affecting insulin therapy perception. Psychological factors such anxiety, insulin fear, and emotional wellness that could impact patient attitudes were not assessed. While the total sample size satisfied the a priori power calculation, the unequal distribution between groups—particularly the smaller prediabetes subgroup—may have limited the power to detect small intergroup differences. Therefore, non-significant findings in subgroup analyses should be interpreted with caution.

The study also has advantages. 160 individuals were enough to reveal notable group differences and ensure the study’s validity, increasing statistical power. There is no study in the literature comparing the perception of T1DM, T2DM and prediabetes patients towards insulin treatment. Including these three groups increases our understanding of insulin perceptions at different diabetes stages. Understanding the physiological and clinical aspects impacting perceptions of insulin therapy, particularly in T1DM and T2DM, is enhanced by the study’s investigation of the link between C-peptide levels and ITAS scores.

## Conclusion

This study highlights the significant differences in perceptions of insulin therapy among patients with prediabetes, T1DM, and T2DM. Individuals with T1DM demonstrated more positive perceptions towards insulin treatment compared to those with T2DM and prediabetes, likely due to their familiarity with the treatment and longer disease duration. On the other hand, individuals with prediabetes expressed comparable unfavorable opinions to those with T2DM, highlighting the necessity of early intervention to stop the development of diabetes and enhance treatment compliance.

Perceptions of insulin treatment were influenced by gender, education, insulin use, and blood glucose monitoring. This suggests that patient education and periodic monitoring may influence views. The study also showed how important sociodemographic variables and clinical criteria like C-peptide levels and disease duration are when assessing patient perceptions.

Our findings highlight the importance of understanding patients’ perceptions of insulin therapy in order to improve treatment acceptance and adherence. Addressing insulin-related attitudes early in the course of the disease—particularly during the prediabetic stage—may help frame insulin therapy as a manageable and potential future treatment option, thereby reducing psychological resistance and minimizing the risk of treatment inertia if insulin later becomes clinically necessary. Incorporating perception-oriented counseling into structured diabetes education programs may therefore enhance long-term treatment acceptance and overall diabetes management, especially among individuals with T2DM and prediabetes. Future longitudinal studies are warranted to better elucidate how insulin perceptions evolve over time and to identify additional psychological factors influencing adherence to insulin therapy.

## Data Availability

The datasets used and/or analysed during the current study are available from the corresponding author on reasonable request.

## Declarations

### Approval of the research protocol

N/A.

### Animal studies

N/A.
